# Virulence factor rtx in *Legionella pneumophila*, evidence suggesting it is a modular multifunctional protein

**DOI:** 10.1186/1471-2164-9-14

**Published:** 2008-01-14

**Authors:** Giuseppe D'Auria, Núria Jiménez, Francesc Peris-Bondia, Carmen Pelaz, Amparo Latorre, Andrés Moya

**Affiliations:** 1 Instituto Cavanilles de Biodiversidad y Biologia Evolutiva, Universitat de València, Spain; 2 CIBER en Epidemiología y Salud Pública (CIBERESP), Spain; 3 National Centre of Microbiology, Institute of Health Carlos III, Majadahonda, Madrid, Spain

## Abstract

**Background:**

The repeats in toxin (Rtx) are an important pathogenicity factor involved in host cells invasion of *Legionella pneumophila *and other pathogenic bacteria. Its role in escaping the host immune system and cytotoxic activity is well known. Its repeated motives and modularity make Rtx a multifunctional factor in pathogenicity.

**Results:**

The comparative analysis of *rtx *gene among 6 strains of *L. pneumophila *showed modularity in their structures. Among compared genomes, the N-terminal region of the protein presents highly dissimilar repeats with functionally similar domains. On the contrary, the C-terminal region is maintained with a fashionable modular configuration, which gives support to its proposed role in adhesion and pore formation. Despite the variability of *rtx *among the considered strains, the flanking genes are maintained in synteny and similarity.

**Conclusion:**

In contrast to the extracellular bacteria *Vibrio cholerae*, in which the *rtx *gene is highly conserved and flanking genes have lost synteny and similarity, the gene region coding for the Rtx toxin in the intracellular pathogen *L. pneumophila *shows a rapid evolution. Changes in the *rtx *could play a role in pathogenicity. The interplay of the Rtx toxin with host membranes might lead to the evolution of new variants that are able to escape host cell defences.

## Background

*Legionella pneumophila *is a gram negative, gamma-proteobacteria organism whose natural hosts are amoebae and protozoa. This bacterium can infect humans by inhalation of aerosols [1,2] entering alveolar macrophages causing the well-known, and often lethal, Legionnaires' disease (LD) or Legionellosis. Despite the great number of isolates of *L. pneumophila*, the ones belonging to serogroup 1 are responsible of about 80 to 90% of cases of Legionellosis [3]. The first critical event during infection by *L. pneumophila *involves the macrophages by the action of the type IV secretion system, which prevents the fusion of the phagosome with the lysosome and its acidification [4,5]. It has been demonstrated that these events start very early after the infection [6]. Several mechanisms play an important role in the formation of infection vacuoles. *Legionella *enters the macrophages by vacuoles that are morphologically similar to macropinosomes by an unusual mechanisms called "coiling phagocytosis" [6,7]. The vacuole is immediately surrounded by vesicles and mitochondria and moves toward the endoplasmic reticulum escaping or delaying fusion with the lysosome [8]. At this stage, the vacuoles offer a perfect niche for bacteria to multiply safely away from the lysosome. *Legionella *is also able to mediate the delayed entrance of the cell in apoptosis by modulating the activity of caspase-3 and other effectors [9,10]. In all these stages the Dot/Icm (Defective in organelle trafficking/Intracellular multiplication) system, involved in the formation of type IV secretor machinery, is the main player acting on the transfer of a series of effectors in the host cell [11-14].

One of the first events in the pathogenic cascade is pore formation, which seems to be caused by a toxin belonging to the Rtx family ("repeats-in toxin") [15]. It was demonstrated that the *rtxA *gene in *L. pneumophila *is strictly related to pathogenicity, and its main role involves adherence to the host membranes, thus facilitating all the molecular trafficking of the bacteria during infection processes [16]. In other bacteria like *Bordetella pertussis*, *Escherichia coli *or *Actinobacillus actinomycetemcomitans*, proteins belonging to the Rtx family are also described as effectors of immune cell lyses and its action is often mediated by specific host membrane receptors [17,18]. RtxA in *L. pneumophila *is a large protein (around 7.000 amino acids) with several repeated structures belonging to, at least, three protein family domains. In *L. pneumophila *strains Lens [GenBank:CR628337] and Paris [GenBank:CR628336] a correlation could exists between the number of repeats and greater invasion and virulence properties [19].

In this work, we analyse the mosaic structure of an RtxA toxin from the highly virulent *L. pneumophila *strain 2300/99. It was isolated in Alcoy (Spain) and was retrieved in several outbreaks in 1999 and 2000, during which more than 200 patients were infected, a dozen of whom died. In all cases, transmission was due to aerosol inhalation from out-door installations [20]. The comparative analysis of the *rtx *locus of closely related *L. pneumophila *serogroup 1 strains showed the existence of a long tandem repeated domain of variable copy number and sequence. Furthermore, we have studied similarity and gene-order conservation of genes flanking the *rtx *region, finding remarkably high levels of rearrangements and diversity of the *rtxA *gene as compared to those from flanking regions. This pattern is completely opposite to that found in several strains of *Vibrio cholerae*, a phylogenetically close extracellular pathogen.

## Results

### *rtx *structure

**Figure 1:**
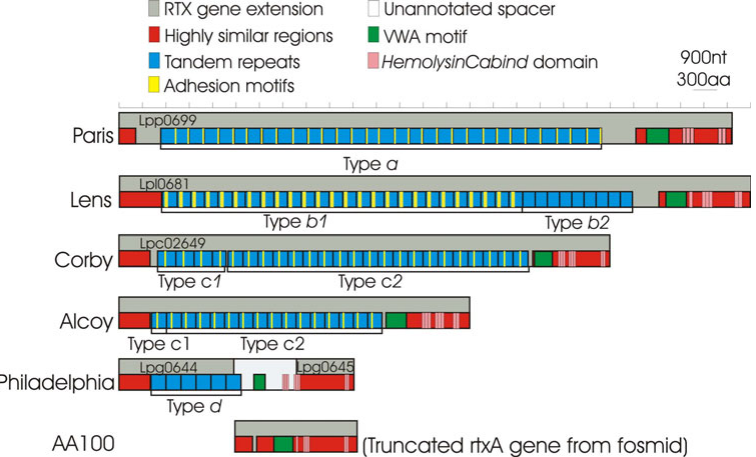
***rtx *structures among the six compared genomes**. Structure of the *rtxA *genes in the six *Legionella *genomes studied. Regions of the *rtx *are marked with correspondent colours (see legend).

Due to the difficulties given by the assembling of such a large and repeated region, the number of repetitions, and consequently, the protein length are approximate and according to what have been published on each released genome. Figure 1 shows the structure of *rtx *region among the different *Legionella *strains and Table 1 is a summary of the main structural characteristics. As it can be observed, *rtx *genes vary in length. The corresponding ORFs are of approximately 7910 aa, 7679 aa, 6289 aa and 4669 aa for strains Lens, Paris Corby and Alcoy (present work), respectively. In the case of the AA100 sequenced contig, two ORFs were identified as previously described by Cirillo *et al*. [21] (*arpB *and *rtxA *fused into one continuous peptide). In the case of the Corby strain, in spite of the high conservation of sequence structure and position of flanking genes, it has been annotated as a "hypothetical protein" and its arrangement is complementary and reversed with respect to Paris, Lens and Philadelphia strains. In *L. pneumophila *Philadelphia, the *rtx *gene is broken into two ORFs (lpg0644 and lpg0645) with an unannotated gap of about 2.600 nt in 3' with respect to the lpg0644. The initial region of *rtx *gene is highly conserved in the five strains (red bars in Figure 1). However, in the strains analyzed in the present work, the region is followed by a variable number of tandem repeats. The repeats contain domains involved in host-membrane interaction, with a wide variability either in copy number and, surprisingly, in nucleotide composition (see Additional file 1). These repeats ranged from 549 nt in the Paris strain to 460 nt in the Lens strain. In the Paris strain, 30 type *a *repeats were described, while in the Lens strain two kinds of repeats, namely *b1 *and *b2*, were observed (as reported also by Cazalet *et al*. [19]). Their sequences differ completely, with 26 and 9 repetitions respectively and with no possible alignment between them (Table 1; Additional file 1, section B). In the Corby strain, we distinguish two different types of repeats, which can be aligned: four named *c1 *and twenty-one named *c2*. In the Alcoy strain, the first repeat spanning from position (nt) 1630 to 2117 is identical to *c1 *type, whereas the other 15 repeats are almost identical to the *c2 *Corby type. Finally, 6 repeats of type *d *were found in the Philadelphia strain. The Philadelphia repeats of the *rtx *gene were identified along and ahead of the ORF lpg0644. Finally, in the case of the AA100 strain, the analysed sequence was not covering the C-terminal region containing the repeats, probably because the studied fosmid insert did not contain the region.

**Table 1:** *rtxA *structure summary. The columns report respectively: strain with accession number in parenthesis; GenBank locus tag used to identify the gene; length in aminoacids; number and types of repeat and number and kind of domains identified by PFAM search.

**Strain**	**Locus tag**	Length (aa)*	Repeats*	**Domains at N-terminal region**
Lens (CR628337)	lpl0681	7910	26 (type *b1*), 9 (type *b2*)	26 Chlam_PMP --- 1 VWA 6 HemolysinCabind
Paris (CR628336)	lpp0699	7679	30 (type *a*)	30 TSP_3 1 VWA 5 HemolysinCabind
Corby (CP000675)	lpc2649	6289	4(type *c1*) 21(type *c2*)	25 HIM 1 VWA 8 HemolysinCabind
Alcoy (EU054322)	lpa00614	4669	16 (type *c*)	16 HIM 1 VWA 8 HemolysinCabind
Philadelphia (AE017354)	lpg0644 spacer lpg0645	1487 865 (presumed) 681 3033 (total)	6 (type *d*)	1 VWA 8 HemolysinCabind
AA100 (AF057703)		1208	---	1VWA 6 HemolysinCabind

*Length of proteins and number of repeats estimated according to the sequences published in relative Genome Project.

After searching in the PFAM database, several kinds of adhesion related domains were identified as part of repeats type *a *(Paris), *b1 *(Lens), *c1 *and *c2 *(Corby and Alcoy). No domains related to adhesion were identified in *rtx *gene of the Philadelphia strain, while only the *c1 *and *c2 *repeats were phylogenetically related (see Additional file 1). Therefore, the way we approached the modular structure of the repeats was by looking at the function of the domains involved in adhesion, with only one exception: the AA100 sequence which spans the region located after the repeats, so it was not possible to include it in our description.

In the four completed genomes (Paris, Lens, Philadelphia and Corby) as well as in the contigs sequenced belonging to the Alcoy and AA100 strains, a von Willebrand factor type A domain (VWA) was identified, subsequent to the regions with tandem repeats. In addition, several blocks of tandem repeats identified as HemolysinCabind domains were also found. These blocks were formed by a number of different repeats: 3+2 repeats in Paris, 1+3+2 in Lens, 3+3+2 in Corby, 3+3+2 in Alcoy, 3+3+2 in Philadelphia and 1+3+2 in AA100. The latter domain was previously described and considered responsible for the virulent activity of the RtxA protein [16,22-24]. A summary of all these structural features of the *rtx *present in different *Legionella *strains is shown in Table 1.

### Comparative genomics and phylogenetic analyses

**Figure 2:**
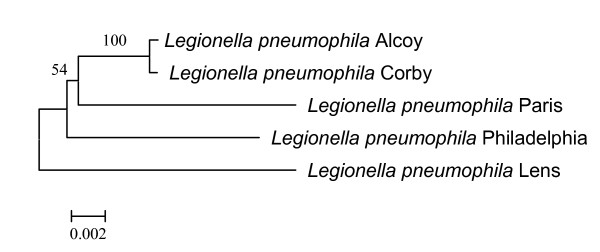
**MLST phylogenetic tree**. Unrooted MLST tree. Numbers at node positions indicate bootstrap values greater than 50% (500 replicates).

**Figure 3:**
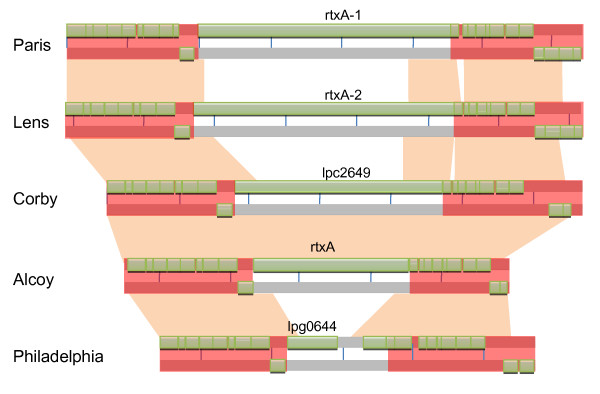
**Plot of *rtx *region of *Legionella *strains**. Comparative plot describing similarity between *rtxA *regions among five *L. pneumophila *genomes. In pink are described regions sharing a nucleotide similarity higher than 70%. Green boxes represent gene positions and strand. Rtx 5' and 3' regions are shaded in red. For an easy visualization, the Corby sequence was complemented and reversed. For a more detailed view see additional file 2

The Multi Locus Sequence Typing (MLST) analysis carried out following the scheme suggested for *L. pneumophila *serogroup 1 [25], using the information available for the five genomes shows that Corby and Alcoy strains are close related, while, due to the low bootstrap values, the positioning of an ancestor to these two strains is not univocal (Figure 2). The whole genome alignments (data not shown), indicate that the genome back-bone is generally maintained with a high level of synteny. However, the synteny surrounding the *rtx *region found in *Legionella *(see Figure 3 and Additional file 2) has not been found in other pathogenic strains. Thus, we performed the same kind of alignment on *Vibrio cholerae *by choosing five *rtx *genes from two complete genomes plus three partial shotgun contigs (see Material and Methods and Additional file 3). As it can be observed, there are slight differences in the organization of *rtx *flanking genes (red shaded zones). Moreover, contrary to what observed in *Legionella*, there is a high level of conservation in the *rtx *region.

Strains Corby and Alcoy, on the other hand, are quite similar in their *rtx *gene at both protein domains and nucleotide level (Figure 1). Similarly to the other *Legionella *genomes, flanking genes are highly conserved both in order and orientation. N-terminal repeats are phylogenetically closely related (see Additional file 1, section C1+C2).

The alignment of repeated domains of the genomes considered here is practically impossible at nucleotide level, and even at amino acid level it is very complicated. Additional file 4 shows the amino acid alignment for each kind of repeat using CLUSTLALW and corrected by eye. The domains identified by PFAM are highlighted. It is not possible to identify any phylogenetic relationships either among the repeats or looking at the sole adhesion domains.

## Discussion

Here we report a comparative analysis of the Rtx toxin, as well as a fine analysis of repeats, identified in this protein in strains of *L. pneumophila *serogroup 1 from four completed genomes (strains Lens, Paris, Philadelphia and Corby), one shotgun ongoing sequencing project (strain Alcoy 2300/99) and one contig coming from a cosmid of the *Legionella *strain AA100. All these strains are known to be virulent [19,21,26,27].

The *rtx *genes analyzed in the six genomes studied, present modularity. The toxin appears to be clearly divided in two regions, the N-terminal, involved in adhesion, and the C-terminal region, involved in adhesion and pore formation in the host membranes. The repeats in the N-terminal region analysed by PFAM database searches, have different kinds of adhesion domains. In all the N-terminal type *a *repeats of the Paris strain, similarity was found with the "Thrombospondin type 3 repeat" (TSP_3) of the human endothelial cell. In humans, this domain has been shown to bind fibrinogen, fibronectin, laminin and type V collagen [28,29]. In the Lens strain, repetitions of type *b1 *show domains similar to the "Chlamydia polymorphic membrane protein" (Chlam_PMP). This obligate intracellular human pathogen causes infection of the upper and lower respiratory tract but the role of this membrane protein is still unknown [30]. No similarity with specific domains was found for *b2 *type repeat of the Lens strain. The last kind of repeat-associated domains, identified in type *c1 *and *c2 *of Alcoy and Corby strains, were similar to the "Haemagglutinin" (HIM) domain that was found in invasins and hemagglutinins, and is associated with the Hep_Hag repeats [31].

Two other types of domains were commonly identified in all the *rtx *genes: the VWA domain involved in adhesion processes [32,33], and another tandem repeated domain related to cytotoxic activity, the haemolysin calcium-binding (HemolysinCabind) site. The latter domain commonly brings a nonapeptide that is related to the adhesion with other host cell surfaces or vacuole membranes and pores formation [23,34].

Except for Alcoy and Corby, which are very similar, the N-terminal repeats and their modular structure are highly variable among strains. Despite these differences, the flanking genes at 5' and 3' are highly conserved in sequence and order, which suggests that *rtx *undergoes a particular intra-genic evolution. Various examples of large non-interspaced repeats within CDS (Coding DNA Sequences) regions were described in bacteria such as *E. coli *and *Bacillus subtilis*, where recombination events were used to explain the distribution of large repeats among genomes [26,35]. The case of *rtx *gene in *L. pneumophila *is particularly interesting due to the high number of observed intra-genic repeats. The origin of these repeats is yet unknown, as no similar sequences (by BLAST searches) have been identified in published data. As described in gene conversion models, DNA can enter to become part of a given gene [36] and afterwards, concerted evolution could be responsible for promoting the different adhesion domains. In fact, although the repeats type *a, b, c*, and *d *do not show any evolutionary relationships, the presence of different adhesion domains points towards a functional evolutionary advantage.

In *V. cholerae *the configuration of the *rtx *gene and its flanking regions is extremely different from that observed in *L. pneumophila*. Comparative analyses of *rtx *gene among five strains show a high level of conservation and the genes located at 5' of *rtx*, are strictly conserved, both in sequence and order, whereas the synteny, and sometimes nucleotide similarity, of those located at 3' do not follow the same pattern (see Additional file 3).

## Conclusion

In *Legionella spp*. it was previously thought that only strains containing an active *rtxA *gene were able to produce infection in humans, and that mutants with a frame-shift inactivating rtxA protein were reduced for entry into host cells and pore formation in host membranes [25]. *rtxA *seems to play a relevant role in the pathogenic activity of *Legionella*, although it also depends on the particular type of host. The variety of repeats and its homogeneous nature at *rtxA *N-terminal region of virulent strains of *L. pneumophila *seems to be acquired by the two mechanisms involved in concerted evolution: intra-genic gene conversion and/or unequal crossing over. As previously described for similar models in other organisms [36,37], these mechanisms can be an important source of creating antigenic variability in *Legionella*, affording the ability to increase the host range and escape from the host's immune defences.

## Methods

### Bacterial strains

*L. pneumophila *serogroup 1 strain (numbered as 2300/99) was isolated from sputum of a patient with Legionnaires' disease and associated to LD outbreak detected in Alcoy (Alicante, Spain) in 1999, from hereinafter strain Alcoy.

### DNA treatments library construction and sequencing

DNA from the *L. pneumophila *Alcoy was extracted as described in Ausubel *et al.*[38] at "Centro Nacional de Microbiología del Instituto de Salud Carlos III (Majadahonda, Madrid, Spain)". Briefly, DNA was sheared by sonication for the construction of two libraries for inserts ranging from 1 to 2 kb and from 2 to 10 kb. Fragments were separated by "Pulsed Field Gel Electrophoresis" applying following conditions: Voltage, 2 V/cm; initial switch time, 0.1 s; final switch time, 1 s; temperature, 14°C; run time, 22 h; angle, 120°. After cutting bands out for the corresponding sizes, DNA was recovered from agarose by electroelution and subsequently purified by phenol/chloroform purification. Ends of fragments were flushed and tailed for TA cloning with "Single dA™ Tailing Kit" (Novagen, #69282-3). After flushing and tailing, DNA was precipitated with 2 Vol of ethanol 96% and 0.1 Vol of sodium acetate 3 M, and eluted in dH_2_O. Next, fragments were cloned with "TOPO XL PCR Cloning Kit" (Invitrogen, #K4700-10). Plasmid DNA purification was done with a "Montage Plasmid Miniprep 96 kit" (Millipore, #LSKP09624) in a "MULTIPROBE II-Robot Liquid Handling System". Sequencing reactions were carried out by the "ABI PRISM Big Dye Terminator v3.0 Ready Reaction Cycle Sequencing Kit" (Applied Biosystems, #4336919) and solved by the "3730XL Genetic Analyzer" (Applied Biosystems).

### Sequences analysis

Sequences used in this paper are: *L. pneumophila *strains Paris [GenBank:CR628336], Lens [GenBank:CR628337], Corby [GenBank:CP000675], Alcoy [GenBank:EU054322], Philadelphia [GenBank:AE017354], AA100 [GenBank:AF057703]. *Vibrio cholerae *strains are: O395 [GenBank:NC_009457], O1 biovar eltor strain N16961 [GenBank:NC_002505], NCTC 8457 [GenBank:AAWD01000018], MZO-3 [GenBank:AAUU01000029] and MO10 [GenBank:DS179636].

The shotgun sequences assembly of the ongoing sequencing project of *L. pneumophila *Alcoy was carried out by the "Staden Package release v1.6.0" [39]. ORF prediction of the yet unfinished strain Alcoy was performed by the use of "Glimmer 3" [40]. Sequence manipulation and annotation was done with "Artemis" software [41]. Comparative analyses were carried out by "BLAST" (Basic Local Alignment Search Tool) suite [42] and the visualization of the results obtained by means of the "ACT" (Artemis Comparative Tool) [43]. Repetitions were identified by the software "Tandem Repeats Finder" [44]. Finally, domain identification was carried out using the PFAM database [45].

### MLST analysis

For this analysis we selected three house keeping genes (*acn*, aconitate hydratase; *groEL*, 60 kDa chaperonin; *recA*, DNA recombination protein) and three fast evolving genes (*flaA*, flagellin; *proA*, zinc metalloprotease; *mompS*, major outer membrane protein) [25]. The nucleotide sequences of each gene were concatenated and a tree based on Maximum Likelihood using Transition-Transversion plus Gamma substitution (as suggested by ModelTest analysis [46]) was obtained by the MEGA3.1 [47] software. Reliability of the monophyletic groups was tested by a bootstrap test with 500 replicates.

### Alcoy strain accession numbers

The genome project of *Legionella pneumophila 2300/99 *Alcoy is maintained at NCBI with project ID 18743. The new Alcoy sequences used in this work are available in GenBank: *rtx *gene contig, accession number EU054322. The genes used for MLST analysis have the following accession numbers: EU221241 (*acn*), EU221242 (*flaA*), EU221243 (*groEL*), EU221244 (*mompS*), EU221245 (*proA*), EU221246 (*recA*).

## Authors' contributions

GD participated in conception, genome assembly/study and drafted the manuscript. NJ and FPB participated in sequencing and genome assembly. CP maintained the studied strain and provided genomic DNA for the proposed work. AL participated in the conception and design of the study and critically revised the manuscript. AM participated in the conception and design of the study and critically revised the manuscript. All authors have read and approved the final manuscript.

## Supplementary Material

Additional file 1 **Nucleotide alignment of repeats**. Alignment of each repeats from each genome analysed. The name of each sequence start with "rep" followed by three letters indicating the strain: lpp, lpl, lpc, lpa, lpg respectively for Paris, Lens, Corby, Alcoy and Philadelphia strains. For each repetition, after the name of the strain is reported the nucleotide position in the respective rtxA protein. "." indicate identical positions; "~" indicates gaps in the alignment. Gray background represents PFAM recognized domains with respective definitions. Click here for file

Additional file 2 **Detailed plot of *rtx *region of *Legionella *strains**. Comparative plot describing similarity between *rtxA *regions among the five *L. pneumophila *genomes. In pink are described regions sharing a nucleotide similarity higher than 70%. Green boxes represent gene positions and strand. Chromosome relative positions are reported for each genome in the central strip. Rtx 5' and 3' regions are shaded in red. For an easy visualization, the Corby sequence was complemented and reversed. Click here for file

Additional file 3 **Detailed plot of *rtx *region of *Vibrio *strains**. Comparative plot describing similarity between *rtxA *regions among the five *Vibrio cholerae *genomes reported in the text. In pink are described regions sharing a nucleotide similarity higher than 70%. Green boxes represent gene position and strand. Chromosome relative positions are reported for each genome in the central strip. Red shadows highlight 5' and 3' flanking regions. Gene names or locus tags are reported for each gene. Click here for file

Additional file 4 **Aminoacids alignment of repeats**. Aminoacids alignment of each kind of repeats. The alignment was obtained by CLUSTALW and corrected by eye. On the left side the name of strain and type of repeat is reported. "~" indicates gaps in the alignment. Colour shaded regions represents domains identified by PFAM searches. Click here for file
