# Supplementary material for: Virulence factor rtx in Legionella pneumophila, evidence suggesting it is a modular multifunctional protein
Source: BMC Genomics. 2008 Jan 14;9:14. doi: 10.1186/1471-2164-9-14 (PMC2257941; doi:10.1186/1471-2164-9-14)
Supplement: Additional file 1 — Nucleotide alignment of repeats. Alignment of each repeats from each genome analysed. The name of each sequence start with "rep" followed by three letters indicating the strain: lpp, lpl, lpc, lpa, lpg respectively for Paris, Lens, Corby, Alcoy and Philadelphia strains. For each repetition, after the name of the strain is reported the nucleotide position in the respective rtxA protein. "." indicate identical positions; "~" indicates gaps in the alignment. Gray background represents PFAM recognized domains with respective definitions. [file 1471-2164-9-14-S1.DOC]

A: *Legionella pneumophila* strain Paris (repetitions type *a*)

10 20 30 40 50 60 70 80 90 100 110 120 130 140 150 160 170 180 190 200

....|....|....|....|....|....|....|....|....|....|....|....|....|....|....|....|....|....|....|....|....|....|....|....|....|....|....|....|....|....|....|....|....|....|....|....|....|....|....|....|

rep lpp_0699 1567:2115 .................................................................................................................a......................................................................................

rep lpp_0699 2116:2664 .......................................................................................................................c................................................................................

rep lpp_0699 2665:3213 .....................................................................c....ga.....................................a......................................................................................

rep lpp_0699 3214:3762 .....................................................................c....ga.....................................a......................................................................................

rep lpp_0699 3763:4311 .......................................................................................................................c................................................................................

rep lpp_0699 4312:4860 tttgaagacgacggccctgtggttgatatggcggtgaaggcaggggctgccttgacgcttgatgaaacgaaaggagtgaaagccggcgatgcgaatgcgaacgacgaagcggcttctgctgatgccaatgacattggttatgcgaagcttgtcggcagtgatttgtttactttaacgaaagacgcaggctctgacgggga

rep lpp_0699 4861:5409 .................................................................................................................a......................................................................................

rep lpp_0699 5410:5958 .......................................................................................................................c................................................................................

rep lpp_0699 5959:6507 ........................................................................................................................................................................................................

rep lpp_0699 6508:7056 .....................................................................n....nn.....................n...............n.....n................................................................................

rep lpp_0699 7057:7605 .....................................................................n....nn.....................n...............n.....n................................................................................

rep lpp_0699 7606:8154 .....................................................................n....nn.....................n...............n.....n................................................................................

rep lpp_0699 8155:8703 .....................................................................n....nn.....................n...............n.....n................................................................................

rep lpp_0699 8704:9252 .....................................................................n....nn.....................n...............n.....n................................................................................

rep lpp_0699 9253:9801 .....................................................................n....nn.....................n...............n.....n................................................................................

rep lpp_0699 9802:10350 .......................................................................................................................c................................................................................

rep lpp_0699 10351:10899 .....................................................................n....nn.....................n...............n.....n................................................................................

rep lpp_0699 10900:11448 .......................................................................................................................c................................................................................

rep lpp_0699 11449:11988 .................................................................................................................a......................................................................................

rep lpp_0699 11998:12546 .................................................................................................................a......................................................................................

rep lpp_0699 12547:13095 .................................................................................................g.....................c................................................................................

rep lpp_0699 13096:13644 .......................................................................................................................c................................................................................

rep lpp_0699 13645:14193 .................................................................................................................a......................................................................................

rep lpp_0699 14194:14742 .......................................................................................................................c................................................................................

rep lpp_0699 14743:15291 .......................................................................................................................c................................................................................

rep lpp_0699 15292:15840 .......................................................................................................................c................................................................................

rep lpp_0699 15841:16389 .................................................................................................................a......................................................................................

rep lpp_0699 16390:16938 .....................................................................c....ga.....................................a......................................................................................

rep lpp_0699 16939:17487 .....................................................................c....ga.....................................a......................................................................................

rep lpp_0699 17488:18036 .................................................................................................................a......................................................................................

210 220 230 240 250 260 270 280 290 300 310 320 330 340 350 360 370 380 390 400

....|....|....|....|....|....|....|....|....|....|....|....|....|....|....|....|....|....|....|....|....|....|....|....|....|....|....|....|....|....|....|....|....|....|....|....|....|....|....|....|

rep lpp_0699 1567:2115 n...........................n.....................................n................................n...............................................................n....................................

rep lpp_0699 2116:2664 ..................................................................t.....................................................................................................................................

rep lpp_0699 2665:3213 ..................................................................t.....................................................................................................................................

rep lpp_0699 3214:3762 ............................g......................................................................t....................................................................................................

rep lpp_0699 3763:4311 ..................................................................t.....................................................................................................................................

rep lpp_0699 4312:4860 gcaatccaccctctttaagcttttggtgagtgctccggcttctggtttggtggatacggcaaccaaccaggccatcgtgttgtctgccaatgcgggcggcacggaagtgttgggcaagaacaccaatggggatgtggtgtttaaggttttattaactgccagcaatggggatgtggaagtctttcaataccgtgcgatca

rep lpp_0699 4861:5409 ..................................................................t.....................................................................................................................................

rep lpp_0699 5410:5958 ..................................................................t.....................................................................................................................................

rep lpp_0699 5959:6507 n...........................n.....................................n................................n...............................................................n....................................

rep lpp_0699 6508:7056 n...........................n.....................................n................................n...............................................................n....................................

rep lpp_0699 7057:7605 n...........................n.....................................n................................n...............................................................n....................................

rep lpp_0699 7606:8154 n...........................n.....................................n................................n...............................................................n....................................

rep lpp_0699 8155:8703 n...........................n.....................................n................................n...............................................................n....................................

rep lpp_0699 8704:9252 n...........................n.....................................n................................n...............................................................n....................................

rep lpp_0699 9253:9801 ............................g.....................................t................................................................................................g....................................

rep lpp_0699 9802:10350 ...................................................................................................t...............................................................n....................................

rep lpp_0699 10351:10899 n...........................n.....................................n................................n...............................................................n....................................

rep lpp_0699 10900:11448 ..................................................................t.....................................................................................................................................

rep lpp_0699 11449:11988 ..................................................................t.....................................................................................................................................

rep lpp_0699 11998:12546 ...................................................................................................t...............................................................g....................................

rep lpp_0699 12547:13095 ...................................................................................................t...............................................................g....................................

rep lpp_0699 13096:13644 ..................................................................t................................t...............................................................g....................................

rep lpp_0699 13645:14193 ............................g.....................................t................................................................................................g....................................

rep lpp_0699 14194:14742 ............................g......................................................................t...............................................................g....................................

rep lpp_0699 14743:15291 ..................................................................t................................................................................................g....................................

rep lpp_0699 15292:15840 ..................................................................t................................................................................................g....................................

rep lpp_0699 15841:16389 ...................................................................................................t....................................................................................................

rep lpp_0699 16390:16938 ..................................................................t.....................................................................................................................................

rep lpp_0699 16939:17487 ..................................................................t.....................................................................................................................................

rep lpp_0699 17488:18036 ........................................................................................................................................................................................................

410 420 430 440 450 460 470 480 490 500 510 520 530 540

....|....|....|....|....|....|....|....|....|....|....|....|....|....|....|....|....|....|....|....|....|....|....|....|....|....|....|....|....|....

rep lpp_0699 1567:2115 ........................................................................................n...............................n...n........................

rep lpp_0699 2116:2664 .....................................................................................................................................................

rep lpp_0699 2665:3213 .....................................................................................................................................................

rep lpp_0699 3214:3762 .....................................................................................................................................................

rep lpp_0699 3763:4311 ........................................................................................................................g...g........................

rep lpp_0699 4312:4860 aacacgagaatgccagcgaccacgacgaatccggcgcgggaggcataatagagcgcattcaagccggctctttgaaattggaagtgaccttaaccgacaaagacggcgacagtgcgaaagacgacctggatttgggtcaaatgatgcgc

rep lpp_0699 4861:5409 .....................................................................................................................................................

rep lpp_0699 5410:5958 ........................................................................................................................g...g........................

rep lpp_0699 5959:6507 ........................................................................................n...............................n...n........................

rep lpp_0699 6508:7056 ........................................................................................n...............................n...n........................

rep lpp_0699 7057:7605 ........................................................................................n...............................n...n........................

rep lpp_0699 7606:8154 ........................................................................................n...............................n...n........................

rep lpp_0699 8155:8703 ........................................................................................n...............................n...n........................

rep lpp_0699 8704:9252 ........................................................................................n...............................n...n........................

rep lpp_0699 9253:9801 ........................................................................................t...............................g...g........................

rep lpp_0699 9802:10350 ........................................................................................n...............................n...n........................

rep lpp_0699 10351:10899 .....................................................................................................................................................

rep lpp_0699 10900:11448 .....................................................................................................................................................

rep lpp_0699 11449:11988 ............................................................................................................................................

rep lpp_0699 11998:12546 .....................................................................................................................................................

rep lpp_0699 12547:13095 .....................................................................................................................................................

rep lpp_0699 13096:13644 ........................................................................................................................g...g........................

rep lpp_0699 13645:14193 ........................................................................................t...............................g...g........................

rep lpp_0699 14194:14742 ........................................................................................................................g...g........................

rep lpp_0699 14743:15291 .....................................................................................................................................................

rep lpp_0699 15292:15840 .....................................................................................................................................................

rep lpp_0699 15841:16389 ........................................................................................t...............................g...g........................

rep lpp_0699 16390:16938 .....................................................................................................................................................

rep lpp_0699 16939:17487 ........................................................................................t...............................g...g........................

rep lpp_0699 17488:18036 .....................................................................................................................................................

**TSP_3 Domain**

B1: *Legionella pneumophila* strain Lens (repetitions type *b1*)

**Chlam_PMP Domain**

10 20 30 40 50 60 70 80 90 100 110 120 130 140 150 160 170 180 190 200

....|....|....|....|....|....|....|....|....|....|....|....|....|....|....|....|....|....|....|....|....|....|....|....|....|....|....|....|....|....|....|....|....|....|....|....|....|....|....|....|

rep_lpl0681 1571:2092 AAGACGACGGCCCGAGCATTGACGGCAGCAAGGTGCTGTCTGCCGATGTGTTGACGGTGGATGAAACGAATTTGGGCGCTAACGCGACGGCCAGTTTTGCTGATAACTTTGCCCAGGCCATTGATTTTGGTGAAGACGGGGCAGGCTCTGTGACCTATGCCCTGGTATTGAATGGCAGCAACGTGGGCTCAGGGCTGTAT

rep_lpl0681 2093:2614 ..................................................C.......................................................T.............................................................................................

rep_lpl0681 2615:3136 ..................................................C.......................................................T.............................................................................................

rep_lpl0681 3137:3658 ........A.........................................C.......................................................T.............................................................................................

rep_lpl0681 3659:4180 ..................................................C.......................................................T.............................................................................................

rep_lpl0681 4181:4702 ..................................................C.......................................................T.............................................................................................

rep_lpl0681 4703:5224 ..................................................C.......................................................T.............................................................................................

rep_lpl0681 5225:5746 ..................................................C...........................TC..T.......................T.............................................................................................

rep_lpl0681 5747:6268 ..................................................C.......................................................T.............................................................................................

rep_lpl0681 6269:6790 .......N..........................................N...........................NN..N.......................N.............................................................................................

rep_lpl0681 6791:7312 .......N..........................................N...........................NN..N.......................N.............................................................................................

rep_lpl0681 7313:7834 .......N..........................................N...........................NN..N.......................N.............................................................................................

rep_lpl0681 7835:8356 .......N..........................................N...........................NN..N.......................N.............................................................................................

rep_lpl0681 8357:8878 .......N..........................................N...........................NN..N.......................N.............................................................................................

rep_lpl0681 8879:9400 .......N..........................................N...........................NN..N.......................N.............................................................................................

rep_lpl0681 9401:9922 .......N..........................................N...........................NN..N.......................N.............................................................................................

rep_lpl0681 9923:10444 .......N..........................................N...........................NN..N.......................N.............................................................................................

rep_lpl0681 10445:10966 .......N..........................................N...........................NN..N.......................N.............................................................................................

rep_lpl0681 10967:11488 .......N..........................................N...........................NN..N.......................N.............................................................................................

rep_lpl0681 11489:12010 .......N..........................................N...........................NN..N.......................N.............................................................................................

rep_lpl0681 12011:12532 ..................................................C.......................................................T.............................................................................................

rep_lpl0681 12533:13054 ..................................................C.......................................................T.............................................................................................

rep_lpl0681 13055:13576 ..................................................C.......................................................T.............................................................................................

rep_lpl0681 13577:14098 ..................................................C.......................................................T.............................................................................................

rep_lpl0681 14099:14620 .......T..........................................C.......................................................T.............................................................................................

rep_lpl0681 14621:15142 ..................................................C.....................................................................................................................................................

210 220 230 240 250 260 270 280 290 300 310 320 330 340 350 360 370 380 390 400

....|....|....|....|....|....|....|....|....|....|....|....|....|....|....|....|....|....|....|....|....|....|....|....|....|....|....|....|....|....|....|....|....|....|....|....|....|....|....|....|

rep_lpl0681 1571:2092 GCCATTGATAACCTGGATGTGAGTACAGCAGACGGTGACGGCATTGGGCGGGGCGGTGAGATTGTATTAAACCAAAACGGCAATGTGGTGACCGGCAGCCTGGGCGGCGTGGATTATTTTACGATTACCATTGATGAGGCGAGCGGTGAGGTGGTGTTTGAGCAGCTGGCTTCGGTATGGCATGCCAATACGGCGAACCC

rep_lpl0681 2093:2614 .......................C....................C....................................................................................................................C......................G...............

rep_lpl0681 2615:3136 .......................C....................C...........................................................................................................................................................

rep_lpl0681 3137:3658 .......................C....................C...........................................................................................................................................................

rep_lpl0681 3659:4180 ........................................................................................................................................................................................................

rep_lpl0681 4181:4702 ........................................................................................................................................................................................................

rep_lpl0681 4703:5224 .......................C....................C....................................................................................................................C......................G...............

rep_lpl0681 5225:5746 .......................C....................C...........................................................................................................................................................

rep_lpl0681 5747:6268 .......................C....................C....................................................................................................................C......................G...............

rep_lpl0681 6269:6790 .......................N....................N....................................................................................................................N......................N...............

rep_lpl0681 6791:7312 .......................N....................N....................................................................................................................N......................N...............

rep_lpl0681 7313:7834 .......................N....................N....................................................................................................................N......................N...............

rep_lpl0681 7835:8356 .......................N....................N....................................................................................................................N......................N...............

rep_lpl0681 8357:8878 .......................N....................N....................................................................................................................N......................N...............

rep_lpl0681 8879:9400 .......................N....................N....................................................................................................................N......................N...............

rep_lpl0681 9401:9922 .......................N....................N....................................................................................................................N......................N...............

rep_lpl0681 9923:10444 .......................N....................N....................................................................................................................N......................N...............

rep_lpl0681 10445:10966 .......................N....................N....................................................................................................................N......................N...............

rep_lpl0681 10967:11488 .......................N....................N....................................................................................................................N......................N...............

rep_lpl0681 11489:12010 .......................N....................N....................................................................................................................N......................N...............

rep_lpl0681 12011:12532 .......................C....................C....................................................................................................................C......................................

rep_lpl0681 12533:13054 .......................C....................C...........................................................................................................................................................

rep_lpl0681 13055:13576 .......................C....................C...........................................................................................................................................................

rep_lpl0681 13577:14098 .......................C....................C....................................................................................................................C......................G...............

rep_lpl0681 14099:14620 .......................C....................C....................................................................................................................C......................G...............

rep_lpl0681 14621:15142 ............................................C....................................................................................................................C......................................

410 420 430 440 450 460 470 480 490 500 510 520

....|....|....|....|....|....|....|....|....|....|....|....|....|....|....|....|....|....|....|....|....|....|....|....|..

rep_lpl0681 1571:2092 CGACGACCAGTCCGCTTTACAGGCTCTGGCCAACAGTTTGGTGGTTCGGGCCACGGTAGTGGATGCCGACGGCGATCAGGCTGTCCATGATTTGGATGTGAGCCAGGGCGTGTTCCAGGTGA

rep_lpl0681 2093:2614 ..........................................................................................................................

rep_lpl0681 2615:3136 ..........................................................................................................................

rep_lpl0681 3137:3658 ..........................................................................................................................

rep_lpl0681 3659:4180 ..........................................................................................................................

rep_lpl0681 4181:4702 ..........................................................................................................................

rep_lpl0681 4703:5224 ..........................................................................................................................

rep_lpl0681 5225:5746 ..........................................................................................................................

rep_lpl0681 5747:6268 ..........................................................................................................................

rep_lpl0681 6269:6790 ........................................................................N.................................................

rep_lpl0681 6791:7312 ........................................................................N.................................................

rep_lpl0681 7313:7834 ........................................................................N.................................................

rep_lpl0681 7835:8356 ........................................................................N.................................................

rep_lpl0681 8357:8878 ........................................................................N.................................................

rep_lpl0681 8879:9400 ........................................................................N.................................................

rep_lpl0681 9401:9922 ........................................................................N.................................................

rep_lpl0681 9923:10444 ........................................................................N.................................................

rep_lpl0681 10445:10966 ........................................................................N.................................................

rep_lpl0681 10967:11488 ........................................................................N.................................................

rep_lpl0681 11489:12010 ........................................................................N.................................................

rep_lpl0681 12011:12532 ..........................................................................................................................

rep_lpl0681 12533:13054 ..........................................................................................................................

rep_lpl0681 13055:13576 ..........................................................................................................................

rep_lpl0681 13577:14098 ..........................................................................................................................

rep_lpl0681 14099:14620 ..........................................................................................................................

rep_lpl0681 14621:15142 ........................................................................T.................................................

B2: *Legionella pneumophila* strain Lens (repetitions type *b2*)

10 20 30 40 50 60 70 80 90 100 110 120 130 140 150 160 170 180 190 200

....|....|....|....|....|....|....|....|....|....|....|....|....|....|....|....|....|....|....|....|....|....|....|....|....|....|....|....|....|....|....|....|....|....|....|....|....|....|....|....|

rep_lpl0681 15167:15625 -CAACGAAACTGACTGGCGTGGTGGATGAAGACGGCCTGGCAGGCGGCATTGCCGGGGGCACAGGCGATGTGGCCGGTCAGGCGGTAGCGGCTTCAGGCAACGTGGCCACCTTGTTCCAAAGCGGAGCGGATGCCCCACTGAGCTATTCCTTGAACCCCAACACCAGCGGCTTGCCGGCTTTAAGTTCCGGTGGCGTGGC

rep_lpl0681 16085:16542 -.......................................................................................................................................................................................................

rep_lpl0681 16544:17001 -.......................................................................................................................................................................................................

rep_lpl0681 17003:17460 -.......................................................................................................................................................................................................

rep_lpl0681 17462:17919 -.......................................................................................................................................................................................................

rep_lpl0681 17921:18378 -.......................................................................................................................................................................................................

rep_lpl0681 18380:18837 -.......................................................................................................................................................................................................

rep_lpl0681 18839:19269 -.......................................................................................................................................................................................................

rep_lpl0681 15625:16083 A.......................................................................................................................................................................................................

210 220 230 240 250 260 270 280 290 300 310 320 330 340 350 360 370 380 390 400

....|....|....|....|....|....|....|....|....|....|....|....|....|....|....|....|....|....|....|....|....|....|....|....|....|....|....|....|....|....|....|....|....|....|....|....|....|....|....|....|

rep_lpl0681 15167:15625 TCTGACTTATGCGGTGTCCGGTGGTACGCTGACGGCGTCTGCAGGCAGCACGCAGGTCTTTACCTTCACGCTCAATGCCAACGGCAATTACACGTTCACCTTGCTGGCGAAGCTCGATCACCCCGCCGGGGCGGATGAAAACGACATTACCATCAATTTGGGCTCTGTCATTAGGGCAACAGACAGCGACGGGGACACGG

rep_lpl0681 16085:16542 ........................................................................................................................................................................................................

rep_lpl0681 16544:17001 ........................................................................................................................................................................................................

rep_lpl0681 17003:17460 ........................................................................................................................................................................................................

rep_lpl0681 17462:17919 ........................................................................................................................................................................................................

rep_lpl0681 17921:18378 ........................................................................................................................................................................................................

rep_lpl0681 18380:18837 ........................................................................................................................................................................................................

rep_lpl0681 18839:19269 ........................................................................................................................................................................................................

rep_lpl0681 15625:16083 ........................................................................................................................................................................................................

410 420 430 440 450 460

....|....|....|....|....|....|....|....|....|....|....|....|

rep_lpl0681 15167:15625 TAGTGGCCGCAGCCGATGGGCTTGTGATTACAGTAGACGACGACACGCCGGTTGCGAGTA

rep_lpl0681 16085:16542 ...........................................................-

rep_lpl0681 16544:17001 ...........................................................-

rep_lpl0681 17003:17460 ..........G................................................-

rep_lpl0681 17462:17919 ...........................................................-

rep_lpl0681 17921:18378 ..................................................A........-

rep_lpl0681 18380:18837 ..........G.......................................A........-

rep_lpl0681 18839:19269 ..........G.....................~~~~~~~~~~~~~~~~~~~~~~~~~~~-

rep_lpl0681 15625:16083 ..........G.......................................A........-

C1+C2: Legionella pneumophila strain Corby and Alcoy (repetitions type c1 and c2)

10 20 30 40 50 60 70 80 90 100 110 120 130 140 150 160 170 180 190 200

....|....|....|....|....|....|....|....|....|....|....|....|....|....|....|....|....|....|....|....|....|....|....|....|....|....|....|....|....|....|....|....|....|....|....|....|....|....|....|....|

rep lpc 1630:2228 GATGCTGGCAGCGACGACGTGGCCAGTCCTTTGGCTTTGTTTGCCGGGGTTAGTCAAAAATCTACGGATATGGCCGGCTTTGCCCAAAGCAGCGGGGCTGTAGTGAGCTCTGCTGGCAGCTTGGTTGGTCAAGACAACGAAGGGGCTACCATTAAATTTTCTTTAGCGATAGCCAATGCAGCGAGCGGATTACAAACCAC

rep lpc 2230:2708 A..........................................................-............................................................................................................................................

rep lpc 2710:3249 ........................................................................................................................................................................................................

rep lpc 3250:3789 .....C..................................................................................................................................................................................................

rep lpc 3790:4326 .....C...........................T.GG......TG..T..GGTCA.C..G........C.....A........G........T..T.....G.....T.....G.....TG..ACG.....G..............C.....C.....C....................G....................

rep lpc 4330:4866 .....C......A...........G....A...T.GG.......G..T..GGTCA.C..G........C.....A........G........T..T.....G.....T.....G.....TG..ACG.....G..............C.....C.....C....................G....................

rep lpc 4870:5406 .....C......A...........G....A...T.GG.......G..T..GGTCA.C..G........C.......................T..T...........T...A.......TG..ACG.....G....................C..........................G....................

rep lpc 5410:5946 .....C......A....................T.GG......TG..T..GGTCA.C..G........C.....A........G........T..T.....G.....T.....G.....TG..ACG.....G..............C.....C.....C....................G....................

rep lpc 5950:6486 .....C......A...........G....A...T.GG.......G..T..GGTCA.C..G........C.....A........G........T..T.....G.....T.....G.....TG..ACG.....G..............C.....C.....C....................G....................

rep lpc 6490:7026 .....C......A...........G....A...T.GG.......G..T..GGTCA.C..G........C.....A........G........T..T.....G.....T.....G.....TG..ACG.....G..............C.....C.....C....................G....................

rep lpc 7030:7566 .....C......A...........G....A...T.GG.......G..T..GGTCA.C..G........C.......................T..T...........T...A.......TG..ACG.....G....................C..........................G....................

rep lpc 7570:8106 .....C......A....................T.GG......TG..T..GGTCA.C..G........C.....A........G........T..T.....G.....T.....G.....TG..ACG.....G..............C.....C.....C....................G....................

rep lpc 8110:8646 .....C......A...........G....A...T.GG.......G..T..GGTCA.C..G........C.....A........G........T..T.....G.....T.....G.....TG..ACG.....G..............C.....C.....C....................G....................

rep lpc 8650:9186 .....C......A...........G....A...T.GG.......G..T..GGTCA.C..G........C.......................T..T...........T...A.......TG..ACG.....G..............C.....C.....C....................G....................

rep lpc 9190:9726 .....C......A...........G....A...T.GG.......G..T..GGTCA.C..G........C.....A........G........T..T.....G.....T.....G.....TG..ACG.....G..............C.....C.....C....................G....................

rep lpc 9730:10266 .....C......A...........G....A...T.GG.......G..T..GGTCA.C..G........C.....A........G........T..T.....G.....T.....G.....TG..ACG.....G..............C.....C.....C....................G....................

rep lpc 10270:10806 .....C......A...........G....A...T.GG.......G..T..GGTCA.C..G........C.......................T..T...........T...A.......TG..ACG.....G....................C..........................G....................

rep lpc 10810:11346 .....C......A...........G....A...T.GG.......G..T..GGTCA.C..G........C.......................T..T...........T...A.......TG..ACG.....G....................C..........................G....................

rep lpc 11350:11886 .....C......A....................T.GG......TG..T..GGTCA.C..G........C.....A........G........T..T.....G.....T.....G.....TG..ACG.....G..............C.....C.....C....................G....................

rep lpc 11890:12426 .....C......A...........G....A...T.GG.......G..T..GGTCA.C..G........C.....A........G........T..T.....G.....T.....G.....TG..ACG.....G..............C.....C.....C....................G....................

rep lpc 12430:12966 .....C......A...........G....A...T.GG.......G..T..GGTCA.C..G........C.......................T..T...........T...A.......TG..ACG.....G..............C.....C.....C....................G....................

rep lpc 12970:13506 .....C......A...........G....A...T.GG.......G..T..GGTCA.C..G........C.....A........G........T..T.....G.....T.....G.....TG..ACG.....G..............C.....C.....C....................G....................

rep lpc 13510:14046 .....C......A...........G....A...T.GG.......G..T..GGTCA.C..G........C.......................T..T...........T...A.......TG..ACG.....G..............C.....C.....C....................G....................

rep lpc 14050:14586 .....C......A...........G....A...T.GG.......G..T..GGTCA.C..G........C.....A........G........T..T.....G.....T...A.......TG..ACG.....G..............C.....C.....C....................G....................

rep lpc 14590:15074 .....C......A...........G....A...T.GG.......G..T..GGTCA.C..G........C.....A........G........T..T.....G.....T.....G.....TG..ACG.....G..............C.....C.....C....................G....................

rep lpa 1630:2117 ........................................................................................................................................................................................................

rep lpa 2170:2709 .....C...........................T.GG......TG..T..GGTCA.C..G........C.....A........G........T..T.....G.....T.....G.....TG..ACG.....G..............C.....C.....C....................G....................

rep lpa 2710:3249 .....C......A...........G....A...T.GG.......G..T..GGTCA.C..G........C.....A........G........T..T.....G.....T.....G.....TG..ACG.....G..............C.....C.....C....................G....................

rep lpa 3250:3789 .....C......A...........G....A...T.GG.......G..T..GGTCA.C..G........C.......................T..T...........T...A.......TG..ACG.....G..............C.....C.....C....................G....................

rep lpa 3790:4329 .....C......A...........G....A...T.GG.......G..T..GGTCA.C..G........C.....A........G........T..T.....G.....T.....G.....TG..ACG.....G..............C.....C.....C....................G....................

rep lpa 4330:4869 .....C......A...........G....A...T.GG.......G..T..GGTCA.C..G........C.......................T..T...........T...A.......TG..ACG.....G....................C..........................G....................

rep lpa 4870:5409 .....C......A....................T.GG......TG..T..GGTCA.C..G........C.....A........G........T..T.....G.....T.....G.....TG..ACG.....G..............C.....C.....C....................G....................

rep lpa 5410:5949 .....C......A...........G....A...T.GG.......G..T..GGTCA.C..G........C.....A........G........T..T.....G.....T.....G.....TG..ACG.....G..............C.....C.....C....................G....................

rep lpa 5950:6489 .....C......A...........G....A...T.GG.......G..T..GGTCA.C..G........C.....A........G........T..T.....G.....T.....G.....TG..ACG.....G..............C.....C.....C....................G....................

rep lpa 6490:7029 .....C......A...........G....A...T.GG.......G..T..GGTCA.C..G........C.......................T..T...........T...A.......TG..ACG.....G....................C..........................G....................

rep lpa 7030:7569 .....C...........................T.GG......TG..T..GGTCA.C..G........C.....A........G........T..T.....G.....T.....G.....TG..ACG.....G..............C.....C.....C....................G....................

rep lpa 7570:8109 .....C......A...........G....A...T.GG.......G..T..GGTCA.C..G........C.....A........G........T..T.....G.....T.....G.....TG..ACG.....G..............C.....C.....C....................G....................

rep lpa 8110:8649 .....C......A...........G....A...T.GG.......G..T..GGTCA.C..G........C.......................T..T...........T...A.......TG..ACG.....G..............C.....C.....C....................G....................

rep lpa 8650:9189 .....C......A...........G....A...T.GG.......G..T..GGTCA.C..G........C.....A........G........T..T.....G.....T.....G.....TG..ACG.....G..............C.....C.....C....................G....................

rep lpa 9190:9729 .....C......A...........G....A...T.GG.......G..T..GGTCA.C..G........C.....A........G........T..T.....G.....T.....G.....TG..ACG.....G..............C.....C.....C....................G....................

rep lpa 9730:10214 .....C......A...........G....A...T.GG.......G..T..GGTCA.C..G........C.....A........G........T..T.....G.....T.....G.....TG..ACG.....G..............C.....C.....C....................G....................

Conserved HIM domain

210 220 230 240 250 260 270 280 290 300 310 320 330 340 350 360 370 380 390 400

....|....|....|....|....|....|....|....|....|....|....|....|....|....|....|....|....|....|....|....|....|....|....|....|....|....|....|....|....|....|....|....|....|....|....|....|....|....|....|....|

rep lpc 1630:2228 GGACGGGGATGCCATCACCTTGACGCTTGAAAGCGGTTTTGTAGTTGGACGAGACGCAGGCGGCGATGCGGTGTTTGCGATTGCCATTGATGCCGATACCGGGGTCTTAAGCCTGGCTCAATACGAATCTATTAAACACCCATCAGGTGGGGCTTCTTATGACGAAGCCGTGGATTTGTCCGGCAAGATCAACGCGGTGG

rep lpc 2230:2708 ........................................................................................................................................................................................................

rep lpc 2710:3249 ........................................................................................................................................................................................................

rep lpc 3250:3789 ........................................................................................................................................................................................................

rep lpc 3790:4326 ...T.................A..C...........CC....G..G..T..T..T..GA.T...A.G..T..TC.G......T.A........T.....G..C..G......G....C...........C..C.........A.C..G..TT.G..C..C..T..G..G...C.CC..GG..................A.

rep lpc 4330:4866 ...T.................A..C...........CC....G..G..T..T..T..GA.T...A.G..T..TC.G......T.A........T.....G..C..G......G.............................A.............................A...........................

rep lpc 4870:5406 ...T.................A..C...........CC....G..G..T..T..T..GA.T...A.G..T..TC.G......T.A........T.....G..C..G......G....C...........C..C.........A.C..G..TT.G..C..C..T..G..G...C.CC..GG............T.....A.

rep lpc 5410:5946 ...T.................A..C...........CC....G..G..T..T..T..GA.T...A.G..T..TC.G......T.A........T.....G..C..G......G....C........................A.C..G..TT.G..C..C..T..G..G...C.CC..GG............T.....A.

rep lpc 5950:6486 ...T.................A..C...........CC....G..G..T..T..T..GA.T...A.G..T..TC.G......T.A........T.....G..C..G......G....C...........C..C.........A.C..G..TT.G..C..C..T..G..G...C.CC..GG............T.....A.

rep lpc 6490:7026 ...T.................A..C...........CC....G..G..T..T..T..GA.T...A.G..T..TC.G......T.A........T.....G..C..G......G....C........................A.............................A...........................

rep lpc 7030:7566 ...T.................A..C...........CC....G..G..T..T..T..GA.T...A.G..T..TC.G......T.A........T.....G..C..G......G....C...........C..C.........A.C..G..TT.G..C..C..T..G..G...C.CC..GG............T.....A.

rep lpc 7570:8106 ...T.................A..C...........CC....G..G..T..T..T..GA.T...A.G..T..TC.G......T.A........T.....G..C..G......G....C...........C..C.........A.C..G..TT.G..C..C..T..G..G...C.CC..GG..................A.

rep lpc 8110:8646 ...T.................A..C...........CC....G..G..T..T..T..GA.T...A.G..T..TC.G......T.A........T.....G..C..G......G.............................A.............................A...........................

rep lpc 8650:9186 ...T.................A..C...........CC....G..G..T..T..T..GA.T...A.G..T..TC.G......T.A........T.....G..C..G......G....C........................A.C..G..TT.G..C..C..T..G..G...C.CC..GG............T.....A.

rep lpc 9190:9726 ...T.................A..C...........CC....G..G..T..T..T..GA.T...A.G..T..TC.G......T.A........T.....G..C..G......G....C...........C..C.........A.C..G..TT.G..C..C..T..G..G...C.CC..GG..................A.

rep lpc 9730:10266 ...T.................A..C...........CC....G..G..T..T..T..GA.T...A.G..T..TC.G......T.A........T.....G..C..G......G....C...........C..C.........A.C..G..TT.G..C..C..T..G..G...C.CC..GG..................A.

rep lpc 10270:10806 ...T.................A..C...........CC....G..G..T..T..T..GA.T...A.G..T..TC.G......T.A........T.....G..C..G......G....C...........C..C.........A.C..G........................A...........................

rep lpc 10810:11346 ...T.................A..C...........CC....G..G..T..T..T..GA.T...A.G..T..TC.G......T.A........T.....G..C..G......G....C...........C..C.........A.C..G..TT.G..C..C..T..G..G...C.CC..GG............T.....A.

rep lpc 11350:11886 ...T.................A..C...........CC....G..G..T..T..T..GA.T...A.G..T..TC.G......T.A........T.....G..C..G......G....C...........C..C.........A.C..G..TT.G..C..C..T..G..G...C.CC..GG..................A.

rep lpc 11890:12426 ...T.................A..C...........CC....G..G..T..T..T..GA.T...A.G..T..TC.G......T.A........T.....G..C..G......G.............................A.............................A...........................

rep lpc 12430:12966 ...T.................A..C...........CC....G..G..T..T..T..GA.T...A.G..T..TC.G......T.A........T.....G..C..G......G....C...........C..C.........A.C..G..TT.G..C..C..T..G..G...C.CC..GG..................A.

rep lpc 12970:13506 ...T.................A..C...........CC....G..G..T..T..T..GA.T...A.G..T..TC.G......T.A........T.....G..C..G......G.............................A.............................A...........................

rep lpc 13510:14046 ...T.................A..C...........CC....G..G..T..T..T..GA.T...A.G..T..TC.G......T.A........T.....G..C..G......G....C........................A.C..G..TT.G..C..C..T..G..G...C.CC..GG............T.....A.

rep lpc 14050:14586 ...T.................A..C...........CC....G..G..T..T..T..GA.T...A.G..T..TC.G......T.A........T.....G..C..G......G....C...........C..C.........A.C..G..TT.G..C..C..T..G..G...C.CC..GG............T.....A.

rep lpc 14590:15074 ...T.................A..C...........CC....G..G..T..T..T..GA.T...A.G..T..TC.G......T.A........T.....G..C..G......G....C...........C..C.........A.C..G..TT.G..C..C..T..G..G...C.CC..GG............T.......

rep lpa 1630:2117 ........................................................................................................................................................................................................

rep lpa 2170:2709 ...T.................A..C...........CC....G..G..T..T..T..GA.T...A.G..T..TC.G......T.A........T.....G..C..G......G....C...........C..C.........A.C..G..TT.G..C..C..T..G..G...C.CC..GG..................A.

rep lpa 2710:3249 ...T.................A..C...........CC....G..G..T..T..T..GA.T...A.G..T..TC.G......T.A........T.....G..C..G......G.............................A.............................A...........................

rep lpa 3250:3789 ...T.................A..C...........CC....G..G..T..T..T..GA.T...A.G..T..TC.G......T.A........T.....G..C..G......G....C...........C..C.........A.C..G..TT.G..C..C..T..G..G...C.CC..GG..................A.

rep lpa 3790:4329 ...T.................A..C...........CC....G..G..T..T..T..GA.T...A.G..T..TC.G......T.A........T.....G..C..G......G....C........................A.............................A...........................

rep lpa 4330:4869 ...T.................A..C...........CC....G..G..T..T..T..GA.T...A.G..T..TC.G......T.A........T.....G..C..G......G....C...........C..C.........A.C..G..TT.G..C..C..T..G..G...C.CC..GG............T.....A.

rep lpa 4870:5409 ...T.................A..C...........CC....G..G..T..T..T..GA.T...A.G..T..TC.G......T.A........T.....G..C..G......G....C...........C..C.........A.C..G..TT.G..C..C..T..G..G...C.CC..GG..................A.

rep lpa 5410:5949 ...T.................A..C...........CC....G..G..T..T..T..GA.T...A.G..T..TC.G......TTA........T.....G..C..G......G.............................A.............................A...........................

rep lpa 5950:6489 ...T.................A..C...........CC....G..G..T..T..T..GA.T...A.G..T..TC.G......T.A........T.....G..C..G......G.............................A.............................A...........................

rep lpa 6490:7029 ...T.................A..C...........CC....G..G..T..T..T..GA.T...A.G..T..TC.G......T.A........T.....G..C..G......G....C...........C..C.........A.C..G..TT.G..C..C..T..G..G...C.CC..GG............T.....A.

rep lpa 7030:7569 ...T.................A..C...........CC....G..G..T..T..T..GA.T...A.G..T..TC.G......T.A........T.....G..C..G......G....C...........C..C.........A.C..G..TT.G..C..C..T..G..G...C.CC..GG..................A.

rep lpa 7570:8109 ...T.................A..C...........CC....G..G..T..T..T..GA.T...A.G..T..TC.G......T.A........T.....G..C..G......G.............................A.............................A...........................

rep lpa 8110:8649 ...T.................A..C...........CC....G..G..T..T..T..GA.T...A.G..T..TC.G......T.A........T.....G..C..G......G....C........................A.C..G..TT.G..C..C..T..G..G...C.CC..GG............T.....A.

rep lpa 8650:9189 ...T.................A..C...........CC....G..G..T..T..T..GA.T...A.G..T..TC.G......T.A........T.....G..C..G......G....C...........C..C.........A.C..G..TT.G..C..C..T..G..G...C.CC..GG............T.....A.

rep lpa 9190:9729 ...T.................A..C...........CC....G..G..T..T..T..GA.T...A.G..T..TC.G......T.A........T.....G..C..G......G....C...........C..C.........A.C..G..TT.G..C..C..T..G..G...C.CC..GG............T.....A.

rep lpa 9730:10214 ...T.................A..C...........CC....G..G..T..T..T..GA.T...A.G..T..TC.G......T.A........T.....G..C..G......G....C...........C..C.........A.C..G..TT.G..C..C..T..G..G...C.CC..GG............T.......

410 420 430 440 450 460 470 480 490 500 510 520 530 540

....|....|....|....|....|....|....|....|....|....|....|....|....|....|....|....|....|....|....|....|....|....|....|....|....|....|....|....|

rep lpc 1630:2228 TGACAGTGACCGACGGCGATGGGGATGTGGCCACACAAGCGATAGGCATAGGCGATGCGATTGTTTTTGAAGACGACGGGCCTGTTGCTCAAATAGCCGCCACGGGTGTCAAAGTGACTCACGATGAAACCGCCGGTGTG

rep lpc 2230:2708 ...........................................................................................................................................-

rep lpc 2710:3249 ............................................................................................................................................

rep lpc 3250:3789 ............................................................................................................................................

rep lpc 3790:4326 .......................................................CAA.G.G..G.......................G..G..T..G..A..C..G.CG..G..C.T...T........GA.A..C---

rep lpc 4330:4866 ........................................................................................G..G..T..G..A..C..GACG..G..C.T...T........GA.A..C---

rep lpc 4870:5406 .......................................................CAA.G.G..G.......................G..G..T..G..A..C..G.CG..G..C.T...T........GA.A..C---

rep lpc 5410:5946 .......................................................CAA.G.G..G.......................G..G..T..G..A..C..G.CG..G..C.T...T........GA.A..C---

rep lpc 5950:6486 .......................................................CAA.G.G..G.......................G..G..T..G..A..C..G.CG..G..C.T...T........GA.A..C---

rep lpc 6490:7026 ........................................................................................G..G..T..G..A..C..GACG..G..C.T...T........GA.A..C---

rep lpc 7030:7566 .......................................................CAA.G.G..G.......................G..G..T..G..A..C..G.CG..G..C.T...T........GA.A..C---

rep lpc 7570:8106 ........................................................................................G..G..T..G..A..C..G.CG..G..C.T...T........GA.A..C---

rep lpc 8110:8646 ........................................................................................G..G..T..G..A..C..G.CG..G..C.T...T........GA.A..C---

rep lpc 8650:9186 .......................................................CAA.G.G..G.......................G..G..T..G..A..C..G.CG..G..C.T...T........GA.A..C---

rep lpc 9190:9726 .......................................................CAA.G.G..G.......................G..G..T..G..A..C..G.CG..G..C.T...T........GA.A..C---

rep lpc 9730:10266 ........................................................................................G..G..T..G..A..C..GACG..G..C.T...T........GA.A..C---

rep lpc 10270:10806 ........................................................................................G..G..T..G..A..C..GACG..G..C.T...T........GA.A..C---

rep lpc 10810:11346 .......................................................CAA.G.G..G.......................G..G..T..G..A..C..G.CG..G..C.T...T........GA.A..C---

rep lpc 11350:11886 ........................................................................................G..G..T..G..A..C..G.CG..G..C.T...T........GA.A..C---

rep lpc 11890:12426 ........................................................................................G..G..T..G..A..C..G.CG..G..C.T...T........GA.A..C---

rep lpc 12430:12966 ........................................................................................G..G..T..G..A..C..G.CG..G..C.T...T........GA.A..C---

rep lpc 12970:13506 ........................................................................................G..G..T..G..A..C..G.CG..G..C.T...T........GA.A..C---

rep lpc 13510:14046 .......................................................CAA.G.G..G.......................G..G..T..G..A..C..G.CG..G..C.T...T........GA.A..C---

rep lpc 14050:14586 .......................................................CAA.G.G..G.......................G..G..T..G..A..C..G.CG..G..C.T...T........GA.A..C---

rep lpc 14590:15074 .......................................................CAA.G.G..G....................-------------------------------------------------------

rep lpa 1630:2117 ........................................................................................

rep lpa 2170:2709 .......................................................CAA.G.G..G.......................G..G..T..G..A..C..G.CG..G..C.T...T........GA.A..CC.T

rep lpa 2710:3249 ........................................................................................G..G..T..G..A..C..G.CG..G..C.T...T........GA.A..CC.T

rep lpa 3250:3789 ........................................................................................G..G..T..G..A..C..G.CG..G..C.T...T........GA.A..CC.T

rep lpa 3790:4329 ........................................................................................G..G..T..G..A..C..GACG..G..C.T...T........GA.A..CC.T

rep lpa 4330:4869 .......................................................CAA.G.G..G.......................G..G..T..G..A..C..G.CG..G..C.T...T........GA.A..CC.T

rep lpa 4870:5409 ........................................................................................G..G..T..G..A..C..G.CG..G..C.T...T........GA.A..CC.T

rep lpa 5410:5949 ........................................................................................G..G..T..G..A..C..GACG..G..C.T...T........GA.A..CC.T

rep lpa 5950:6489 ........................................................................................G..G..T..G..A..C..GACG..G..C.T...T........GA.A..CC.T

rep lpa 6490:7029 .......................................................CAA.G.G..G.......................G..G..T..G..A..C..G.CG..G..C.T...T........GA.A..CC.T

rep lpa 7030:7569 .......................................................CAA.G.G..G.......................G..G..T..G..A..C..G.CG..G..C.T...T........GA.A..CC.T

rep lpa 7570:8109 ........................................................................................G..G..T..G..A..C..G.CG..G..C.T...T........GA.A..CC.T

rep lpa 8110:8649 .......................................................CAA.G.G..G.......................G..G..T..G..A..C..G.CG..G..C.T...T........GA.A..CC.T

rep lpa 8650:9189 .......................................................CAA.G.G..G.......................G..G..T..G..A..C..G.CG..G..C.T...T........GA.A..CC.T

rep lpa 9190:9729 .......................................................CAA.G.G..G.......................G..G..T..G..A..C..G.CG..G..C.T...T........GA.A..CC.T

rep lpa 9730:10214 .......................................................CAA.G.G..G....................

Type c1, Type c2

rep lpc: *Legionella pneumophila* strain Corby

rep lpa: *Legionella pneumophila* strain Alcoy

Numbers after clone names indicate nucleotide positions with respect to the *rtxA* gene.

D: *Legionella pneumophila* strain Philadelphia (repetitions type *d*)

10 20 30 40 50 60 70 80 90 100 110 120 130 140 150 160 170 180 190 200

....|....|....|....|....|....|....|....|....|....|....|....|....|....|....|....|....|....|....|....|....|....|....|....|....|....|....|....|....|....|....|....|....|....|....|....|....|....|....|....|

rep lpg0644 688113:688650 GACGACGGTCCGAGCGTGACGATGGCGGTGAGCGATAATAATGCGATCACCTTGAACACCCAGGATGCCGATACGATTGGGGCAGCCAGTGACAGCGACAGCGCAAGCTTTGCGGCCGCCTTTGCGGTGACTCCTAATTACGGGGCCGATGGTGCGGGCACGACGGTGACCACTTACGCCTTGAGTGTGTCCGCCCCAAG

rep lpg0644 687576:688112 .............................................................................................................................................................................C......................-...

rep lpg0644 687039:687575 ....................................................................G..G........T.T.CTTTCC....C..............................................................................C......................-...

rep lpg0644 686502:687038 .............................................................................................................................................................................C......................-...

rep lpg0644 685965:686501 .............................................................................................................................................................................C......................-...

rep lpg0644 685428:685964 .............................................................................................................................................................................C......................-...

210 220 230 240 250 260 270 280 290 300 310 320 330 340 350 360 370 380 390 400

....|....|....|....|....|....|....|....|....|....|....|....|....|....|....|....|....|....|....|....|....|....|....|....|....|....|....|....|....|....|....|....|....|....|....|....|....|....|....|....|

rep lpg0644 688113:688650 GGGTGGATTCCGGCCTTGATAACAACGGCAACAACATCTACCTGTACAACATCGCAGGTTCTGTGGTGGGTTCAACATCAGCCACGCAGGCGGGTATTACCACAGGCAACACCATCTTTTCATTGGATGTGAACAGCAGCAGTGGGGTGGTGACTTTAACGCAACACCAGGAAGTGGACCACGGTTTGCCGGGAGCCAGT

rep lpg0644 687576:688112 ........................................................................................................................................................................................................

rep lpg0644 687039:687575 ........................................................................................................................................................................................................

rep lpg0644 686502:687038 ........................................................................................................................................................................................................

rep lpg0644 685965:686501 ........................................................................................................................................................................................................

rep lpg0644 685428:685964 ........................................................................................................................................................................................................

410 420 430 440 450 460 470 480 490 500 510 520 530

....|....|....|....|....|....|....|....|....|....|....|....|....|....|....|....|....|....|....|....|....|....|....|....|....|....|....|...

rep lpg0644 688113:688650 TCCAATTACGCGGCTCAGGAAGCGATATTGAATACGGGCCTGGTCTTTTTAAACGCCACCGCAGTGACGACGGATGGGGATGGGGACACGGCGACTGCCAGTGCGTCTTTGGATTTGGGCGGCAATGTGAAGTTTGAT

rep lpg0644 687576:688112 ..................................................G............................................C..........................................

rep lpg0644 687039:687575 ..................................................G......................................A..C..C..........................................

rep lpg0644 686502:687038 ..................................................G.........................................C..C..........................................

rep lpg0644 685965:686501 ..................................................G......................................A..C..C..........................................

rep lpg0644 685428:685964 ..................................................G............................................C..........................................
