# Supplementary material for: Virulence factor rtx in Legionella pneumophila, evidence suggesting it is a modular multifunctional protein
Source: BMC Genomics. 2008 Jan 14;9:14. doi: 10.1186/1471-2164-9-14 (PMC2257941; doi:10.1186/1471-2164-9-14)
Supplement: Additional file 2 — Detailed plot of rtx region of Legionella strains. Comparative plot describing similarity between rtxA regions among the five L. pneumophila genomes. In pink are described regions sharing a nucleotide similarity higher than 70%. Green boxes represent gene positions and strand. Chromosome relative positions are reported for each genome in the central strip. Rtx 5' and 3' regions are shaded in red. For an easy visualization, the Corby sequence was complemented and reversed. [file 1471-2164-9-14-S2.PPT]

## Slide 1
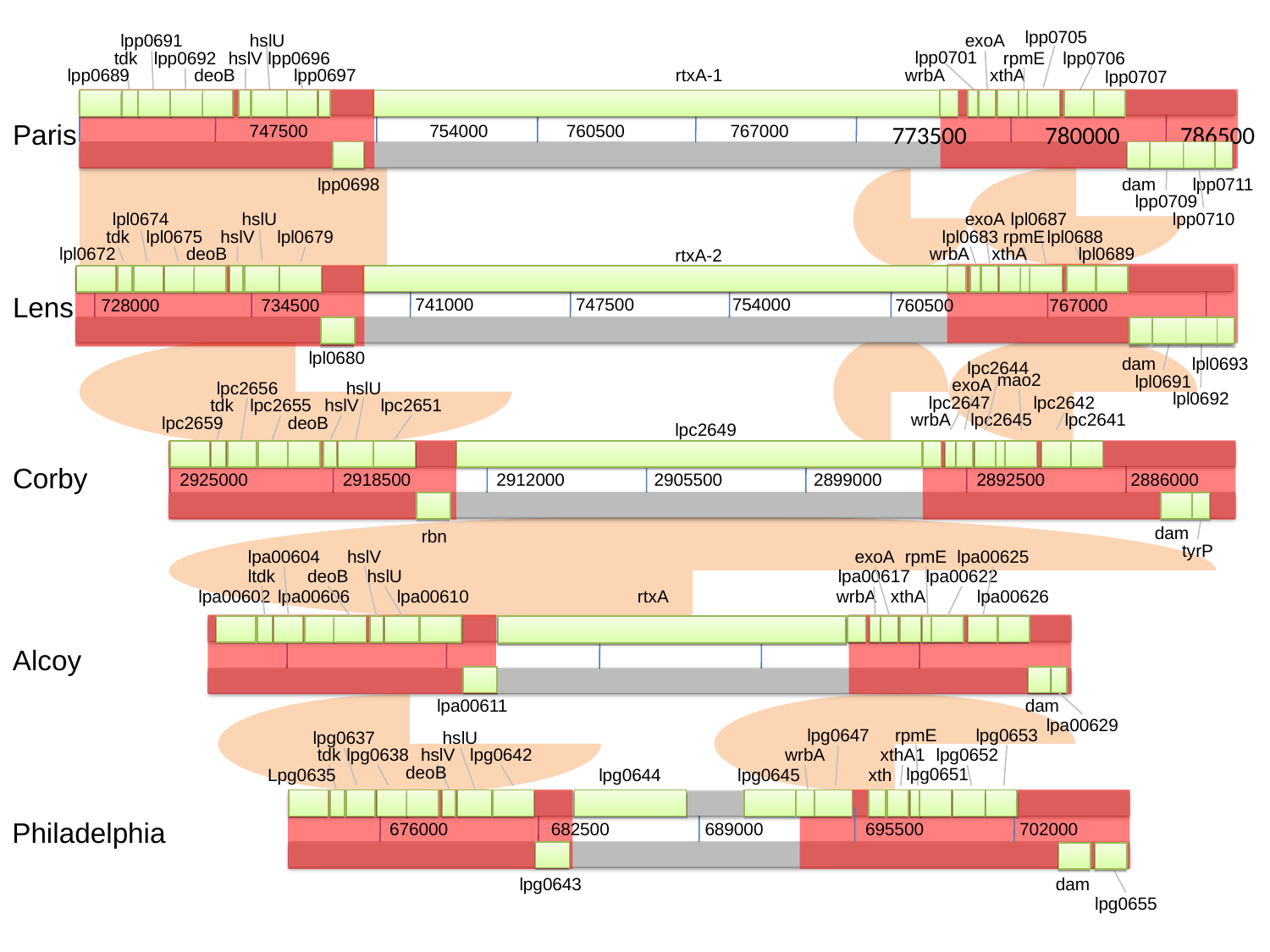

lpp0705
lpp0691
hslU
exoA
lpp0701
tdk
lpp0692
hslV
lpp0696
rpmE
lpp0706
lpp0689
deoB
lpp0697
rtxA-1
wrbA
xthA
lpp0707
747500
773500
780000
786500
Paris
754000
760500
767000
lpp0698
dam
lpp0711
lpp0709
lpp0710
lpl0674
hslU
exoA
lpl0687
tdk
lpl0675
hslV
lpl0679
lpl0683
rpmE
lpl0688
lpl0672
deoB
wrbA
xthA
lpl0689
rtxA-2
728000
734500
760500
767000
Lens
741000
747500
754000
lpl0680
dam
lpl0693
lpc2644
mao2
lpl0691
exoA
lpc2656
hslU
lpl0692
lpc2647
lpc2642
tdk
lpc2655
hslV
lpc2651
wrbA
lpc2645
lpc2641
lpc2659
deoB
lpc2649
2925000
2918500
2892500
2886000
Corby
2912000
2905500
2899000
dam
rbn
tyrP
lpa00604
hslV
exoA
rpmE
lpa00625
ltdk
deoB
hslU
lpa00617
lpa00622
lpa00602
lpa00606
lpa00610
rtxA
wrbA
xthA
lpa00626
Alcoy
lpa00611
dam
lpa00629
lpg0647
rpmE
lpg0653
lpg0637
hslU
tdk
lpg0638
hslV
lpg0642
wrbA
xthA1
lpg0652
deoB
lpg0651
Lpg0635
lpg0644
lpg0645
xth
Philadelphia
676000
682500
689000
695500
702000
lpg0643
dam
lpg0655
