# Supplementary material for: Virulence factor rtx in Legionella pneumophila, evidence suggesting it is a modular multifunctional protein
Source: BMC Genomics. 2008 Jan 14;9:14. doi: 10.1186/1471-2164-9-14 (PMC2257941; doi:10.1186/1471-2164-9-14)
Supplement: Additional file 3 — Detailed plot of rtx region of Vibrio strains. Comparative plot describing similarity between rtxA regions among the five Vibrio cholerae genomes reported in the text. In pink are described regions sharing a nucleotide similarity higher than 70%. Green boxes represent gene position and strand. Chromosome relative positions are reported for each genome in the central strip. Red shadows highlight 5' and 3' flanking regions. Gene names or locus tags are reported for each gene. [file 1471-2164-9-14-S3.PPT]

## Slide 1
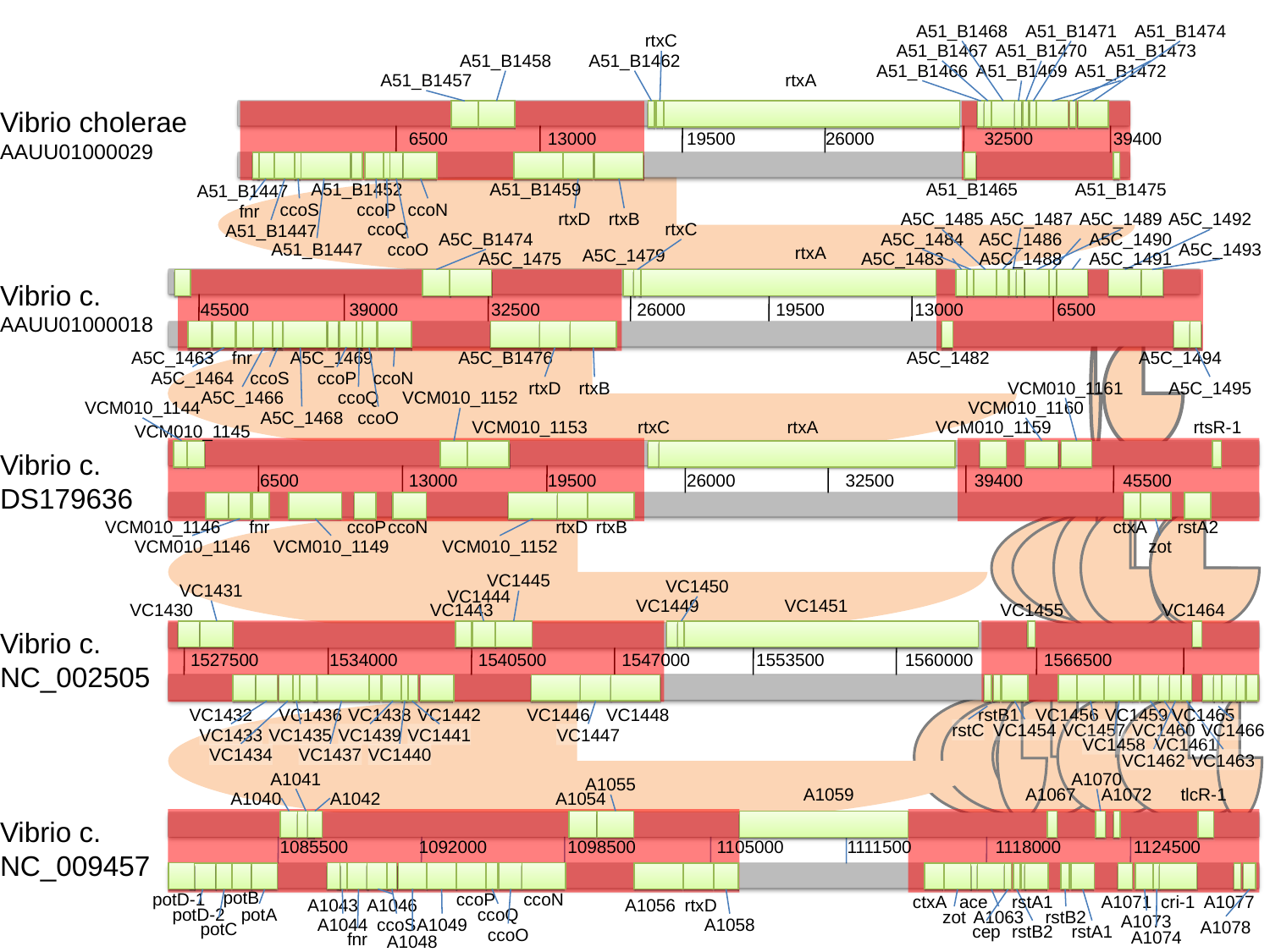

A51_B1468
A51_B1471
A51_B1474
rtxC
A51_B1467
A51_B1470
A51_B1473
A51_B1458
A51_B1462
A51_B1466
A51_B1469
A51_B1472
A51_B1457
rtxA
Vibrio cholerae
AAUU01000029
6500
13000
19500
26000
32500
39400
A51_B1452
A51_B1459
A51_B1465
A51_B1475
A51_B1447
ccoS
ccoP
ccoN
fnr
rtxD
rtxB
A5C_1485
A5C_1487
A5C_1489
A5C_1492
ccoQ
rtxC
A51_B1447
A5C_B1474
A5C_1484
A5C_1486
A5C_1490
A51_B1447
ccoO
A5C_1493
rtxA
A5C_1479
A5C_1475
A5C_1483
A5C_1488
A5C_1491
Vibrio c.
AAUU01000018
45500
39000
32500
26000
19500
13000
6500
A5C_1463
fnr
A5C_1469
A5C_B1476
A5C_1482
A5C_1494
ccoN
A5C_1464
ccoS
ccoP
rtxD
rtxB
VCM010_1161
A5C_1495
A5C_1466
ccoQ
VCM010_1152
VCM010_1144
VCM010_1160
A5C_1468
ccoO
VCM010_1153
rtxC
rtxA
VCM010_1159
rtsR-1
VCM010_1145
Vibrio c.
DS179636
6500
13000
19500
26000
32500
39400
45500
VCM010_1146
fnr
ccoP
ccoN
rtxD
rtxB
rstA2
ctxA
VCM010_1146
VCM010_1149
VCM010_1152
zot
VC1445
VC1450
VC1431
VC1444
VC1449
VC1451
VC1430
VC1443
VC1455
VC1464
Vibrio c.
NC_002505
1527500
1534000
1540500
1547000
1553500
1560000
1566500
rstB1
VC1456
VC1459
VC1465
VC1432
VC1436
VC1438
VC1442
VC1446
VC1448
rstC
VC1454
VC1457
VC1460
VC1466
VC1433
VC1435
VC1439
VC1441
VC1447
VC1458
VC1461
VC1434
VC1437
VC1440
VC1462
VC1463
A1041
A1070
A1055
A1059
A1067
A1072
tlcR-1
A1040
A1042
A1054
Vibrio c.
NC_009457
1085500
1092000
1098500
1105000
1111500
1118000
1124500
potB
potD-1
ccoP
ccoN
ctxA
ace
rstA1
A1071
cri-1
A1077
A1043
A1046
A1056
rtxD
potD-2
potA
ccoQ
zot
A1063
rstB2
A1073
A1044
ccoS
A1049
A1058
A1078
potC
cep
rstB2
rstA1
ccoO
A1074
fnr
A1048
