# Supplementary material for: Virulence factor rtx in Legionella pneumophila, evidence suggesting it is a modular multifunctional protein
Source: BMC Genomics. 2008 Jan 14;9:14. doi: 10.1186/1471-2164-9-14 (PMC2257941; doi:10.1186/1471-2164-9-14)
Supplement: Additional file 4 — Aminoacids alignment of repeats. Aminoacids alignment of each kind of repeats. The alignment was obtained by CLUSTALW and corrected by eye. On the left side the name of strain and type of repeat is reported. "~" indicates gaps in the alignment. Colour shaded regions represents domains identified by PFAM searches. [file 1471-2164-9-14-S4.DOC]

Repetitions alignment of amminoacids translation

10 20 30 40 50 60 70 80 90

....|....|....|....|....|....|....|....|....|....|....|....|....|....|....|....|....|....|

**lpp_a**  **FEDDGPVVDMAVKAGAALTLDETKGVKAGDANANDEAASADANDIGYAKLVGSDLFTLTKDAGSDGEQSTLFK~~~~~~~~~~~~~~~~L**

**lpg_d**  **~~DDGPSVTMAVSDNNAITLNTQDADTIGAASDSDSASFAAA~~~~~~~~~~~~~FAVTPNYGADGAGTTVTT~~~~~~~~~~~~~~~~Y**

**lpl_b1** **~XDDGPSIDG~SKVLSADVLTV~DETNLGANA~~~~~~~~~~~~TASFADNFAQAIDFGEDGAGSVTYALVLNGSNVGSGLYAI~~~~DN**

**lpl_b2** **~~~XTKLTGVVDEDGLAGGIAG~~~~~~GTGDV~~~AGQAVA~~~~~~~~~~~~~~~~~~~~~~~~~~~~ASGNVATLFQSGADAPLS~Y**

**lpc c1** **~~~DAGSDDVASPLALFAGVSQKSTDMAGFAQS~~~SGAVVS~~~~SAGSLVGQDNEGATIKFSLAIANAASGLQTTDGDAITLTL~~~~**

**lpc_c2** **~~~DAGSNDVAGPLSVFAGVVNKSTDMAGFAQS~~~SGAVVS~~~~STGSVTGQDNEGATIKFSLAIANAASGLQTTDGDAITLTL~~~~**

**lpa_c2** **~~~DAGSNDVAGPLSVFAGVVNKSTDMAGFAQS~~~SGAVVS~~~~STGSVTGQDNEGATIKFSLAIANAASGLQTTDGDAITLTL~~~~**

100 110 120 130 140 150 160 170 180

....|....|....|....|....|....|....|....|....|....|....|....|....|....|....|....|....|....|

**lpp_a**  **LVSAPASGLVDTATNQAIVLSANAGGTEVLGKNTNGDVVFKVLLTASNG~~~~~~~DVEVFQYRAIKHENASDHDESGAGGIIERIQAGS**

**lpg_d**  **ALSVSAQGVDSGLDNNGNNIYLYNIAGSVVGSTSATQAGITTGNTIFSLDVNSSSGVVTLTQHQEVDHGLPGASSNYAAQEAILNTGLVF**

**lpl_b1** **LDVSTADGDGIGRGGEIVLNQNGNV~~~~~~~~VTGSLGGVDYFTITIDEASG~~~EVVFEQLASVWHANTANPDDQSALQALANSLVVR**

**lpl_b2** **SLNPNTSGLPALSSGGVALTYAVSGGTLTASAGSTQVFTFTLNANG~~~~~~~~~~~NYTFTLLAKLDHPAGADENDITINLGSVIRATD**

**lpc c1** **~~ES~GFVVGRDAGGDAVFAIAIDADTG~VLSLAQYESIKHPSGGASYDE~~~~~~~~~~~~~~~~~~~~~~~~~~~~~~~~~AVDLSGK**

**lpc_c2** **~~ES~GLVVGRDASGKAVLAISIDADTG~VLSVAQYESIKHPTGGSSYDE~~~~~~~~~~~~~~~~~~~~~~~~~~~~~~~~~AVHLGGK**

**lpa_c2** **~~ES~GLVVC~~~~~~~~~~~~~~~~~~~~~~~~~~~~~~~~~~~~~~~~~~~~~~~~~~~~~~~~~~~~~~~~~~~~~~~~~~~~~~~K**

190 200 210 220 230

....|....|....|....|....|....|....|....|....|....|.

**lpp_a**  **LKLEVTLTDKDGDSAKDDLDLGQMMR~~~~~**

**lpg_d**  **LNATAVTTDGDGDTATASASLDLGGNVKFD**

**lpl_b1** **A~~~~TVVDADGD~QAVHDLDVSQGVFQV~~**

**lpl_b2** **SDGDTVVAAADGL~~~~~~~~~~VITVDDDTPVAS~**

**lpc c1** **INAVVTVTDGDGDVATQAIGIGDAIVFEDDGPVAQIAATGVKVTHDETAGV**

**lpc_c2** **INAVVTVTDGDGDVATQAIGIGDKVVFEDDGPVAQIAATGAKVIHDETTG~**

**lpa_c2** **INAVVTVTDGDGDVATQAIGIGDAIVFEDDGPVAQIAATGAKVIHDETTGL**

Amminoacids alignments of one sequence from each repetition type

Shadowed are represented the adhesion domains Chlam_PMP, HIM and TSP_3, see text for details.
